# Multi-Omics Analysis Reveals the Regulation of Amino Acid Biosynthesis in *Cyclocarya paliurus* Leaves Under Salt Stress

**DOI:** 10.3390/ijms262110444

**Published:** 2025-10-27

**Authors:** Lei Zhang, Kun Hong, Zijie Zhang, Xulan Shang, Shengzuo Fang

**Affiliations:** 1 State Key Laboratory for Development and Utilization of Forest Food Resources, Nanjing Forestry University, Nanjing 210037, China; zhanglei321@njfu.edu.cn (L.Z.);; 2 College of Forestry and Grassland, Co-Innovation Center for the Sustainable Forestry in Southern China, Nanjing Forestry University, Nanjing 210037, China

**Keywords:** wheel wingnut, phenylalanine, transcriptome and metabolome, WGCNA, abiotic stress

## Abstract

Amino acids are essential for plant growth and stress adaptation, and they exhibit distinct patterns under salt stress. However, the biosynthesis and accumulation of amino acids in *C. paliurus* under salt stress have not been fully studied. This research integrated metabolomics and transcriptomics data from *C. paliurus* leaves sampled at four salt concentrations and two sampling times to reveal the mechanisms underlying amino acid metabolism in response to salt stress. Principal component analysis revealed an environmental dependence in the amino acid accumulation patterns, with significant differences in amino acid content observed between samples treated with different salt concentrations and at different time points. Weighted gene correlation network analysis (WGCNA) identified key modules related to amino acid metabolism, with threonine synthase being upregulated under salt stress. Additionally, amino acids, such as proline, which function as osmolytes are increasing. The overexpression of structural genes in the phenylpropanoid biosynthesis pathway and genes encoding phenylalanine ammonia lyase was closely associated with amino acid accumulation, a process regulated by multiple transcription factors. These findings elucidate amino acid-mediated molecular responses to salinity and offer practical guidance for establishing *C. paliurus* plantations in southeastern coastal beach-lands of China.

## 1. Introduction

Salinity is one of the most severe abiotic stresses impacting plant productivity. The salt content of the coastal saline surface soil in Jiangsu Province, China, ranges from 0.20% to 0.50%. [1]. Salt stress is a hypertonic stress that hinders water uptake in plants [2]. It results in the accumulation of Na^+^ and Cl^−^ in the cytosol, and excessive Na^+^ accumulation leads to increased production of reactive oxygen species (ROS), causing oxidative damage to proteins, lipids, and nucleic acids. This damage can significantly reduce crop yield, and in extreme cases, may ultimately result in the demise of the plant [3,4,5]. In response to elevated levels of salt in the environment, plants have evolved a range of adaptations. These include mechanisms such as osmotic regulation and secondary metabolism to mitigate the adverse effects of salinity on plant tissue. [6,7]. In addition, amino acids, as the main components influencing plant flavor, play a significant role in abiotic stress responses. For instance, proline functions to regulate osmotic balance, while tyrosine acts as a biostimulant. [8,9].

*Cyclocarya paliurus* (Batal) IIjinskaja is an endemic plant belonging to the *Cyclocarya* genus of the Juglandaceae family, primarily distributed in the south of China [10]. It is colloquially referred to as the “sweet tea tree” due to the flavor of its leaves. It has also traditionally been utilized as an herbal tea [11]. Pharmacological studies on *C. paliurus* have demonstrated that its leaf extracts exhibit a multitude of biological functions, including hypoglycaemic, hypolipidemic and hypotensive effects. This is attributable to the synergistic effect of a plethora of phytochemicals, such as triterpenoids, flavonoids, polyphenols, amino acid and other compounds [12]. Notably, amino acids have been considered as the main factor to tea flavor and function [13,14]. In order to satisfy the demand for leaves for use in developing value-added products, it is recommended that coastal saline land be used to cultivate *C. paliurus* plantations. Nevertheless, the regulatory mechanisms implicated in the amino acid metabolism of *C. paliurus* leaves under salt stress remain to be the subject of further elucidation.

In addition to the formation of proteins, amino acids can function as independent entities within a range of physiological processes within plant organisms. For instance, proline is one significant osmoregulatory substance that plays a crucial role in maintaining osmotic pressure balance within and outside cells [15]. In addition, GABA, a common non-protein amino acid in plants, is not only a signalling molecule that regulates plant growth and the formation of tolerance, but also has ROS-scavenging activity and reduces the level of lipid peroxides [16,17,18]. Amino acid metabolism tends to vary according to the environmental conditions under which the plant is grown [19]. For example, cadmium pollution has been demonstrated to interfere with amino acid metabolism in plants, resulting in a significant increase in aspartic acid, glutamic acid, asparagine, histidine, and phenylalanine levels in *Capsicum annum* [20]. However, the extent of environmental factors capable of promoting the biosynthesis and subsequent accumulation of amino acids within the leaves of *C. paliurus* remains unknown.

Integrative transcriptome and metabolome analyses have become a significant method for analysing the gene regulation and metabolite accumulation in plants in response to environmental stress [21]. For example, transcriptomic and metabolomic profiling helped to investigate sugar metabolism in response to PEG-induced drought stress in *C. paliurus* seedlings, providing valuable insights for the breeding of drought-tolerant plants [22]. This study combines metabolomic and transcriptomic analyses of salt-stressed *C. paliurus* leaves to address three key goals: (1) to conduct both quantitative and qualitative analyses of amino acids; (2) to investigate the effects of varying sampling times and salt concentrations on amino acid composition and content; and (3) to elucidate the biosynthetic mechanisms of amino acids in the leaves of *C. paliurus* under salt stress. Findings of this study can enhance our understanding of the molecular regulatory mechanisms involved in environmental stress responses and offer a theoretical foundation for the exploitation of salinity in sustainable forestry production.

## 2. Results

### 2.1. Amino Acids Accumulation in C. paliurus Leaves

**Figure 1:**
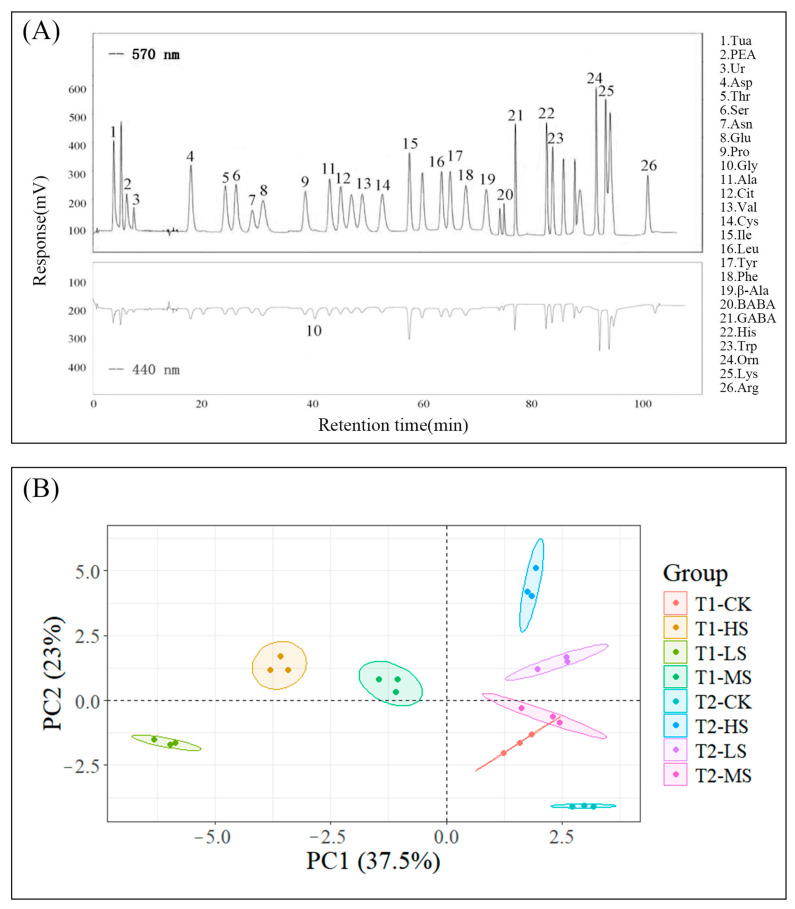
Chromatogram and PCA of amino acids. (**A**) High-performance liquid chromatography (HPLC) of 26 amino acids extracted from *C. paliurus* leaves. (**B**) PCA scatter plot where colors denote different salt stress biological replicates. T1 and T2 denote the two sampling times, which correspond to 15 and 30 days after treatment, respectively. CK, LS, MS and HS indicate the non-, low-, mid-, and high-salinity treatments. T1 and T2 represent two sampling times, which are 15 and 30 days after treatments.

A total of 26 amino acids were successfully detected and characterized by using chromatographic techniques in combination with post-column derivatization (Figure 1A). These included eight essential amino acids (Thr (threonine), Phe (phenylalanine), Leu (leucine), Ile (isoleucine), Val (valine), His (histidine), Trp (tryptophan) and Lys (lysine)) and ten non-essential amino acids (Ser (serine), Asp (aspartic acid), Asn (asparagine), Glu (glutamic acid), Pro (proline), Gly (glycine), Ala (alanine), Cys (cysteine), Tyr (tyrosine) and Arg(arginine)), all of which were consistently detected across *C. paliurus* leaf samples subjected to four levels of NaCl treatment at two time points. Ur(urea), Cys, and Arg were the most abundant amino acids, accounting on average for 37.04%, 19.15%, and 6.48% of the total amino acid content, respectively (Table S1).

Principal component analysis (PCA) revealed distinct metabolic profiles of amino acids under different treatments and times, with PC1 and PC2 explaining 37.5% and 23% of the total variation, collectively explaining variance of 60.5% (Figure 1B). The separation along PC1 primarily distinguished T1 from T2 groups and was driven by differences in Lys (lysine) content, which had the highest loading (0.29) (Table S2), indicating time-dependent metabolic shifts. Meanwhile, PC2 captured differences among the salt stress treatments, influenced by variations in Asn (asparagine) levels, the largest loading factor (−0.34) (Table S2). These results highlight distinct patterns of amino acid reprogramming, with salt treatments clustering separately from controls, providing critical insights into the adaptive mechanisms of amino acid metabolism in response to salt stress.

### 2.2. Transcriptomic Response in C. paliurus Leaves

**Figure 2:**
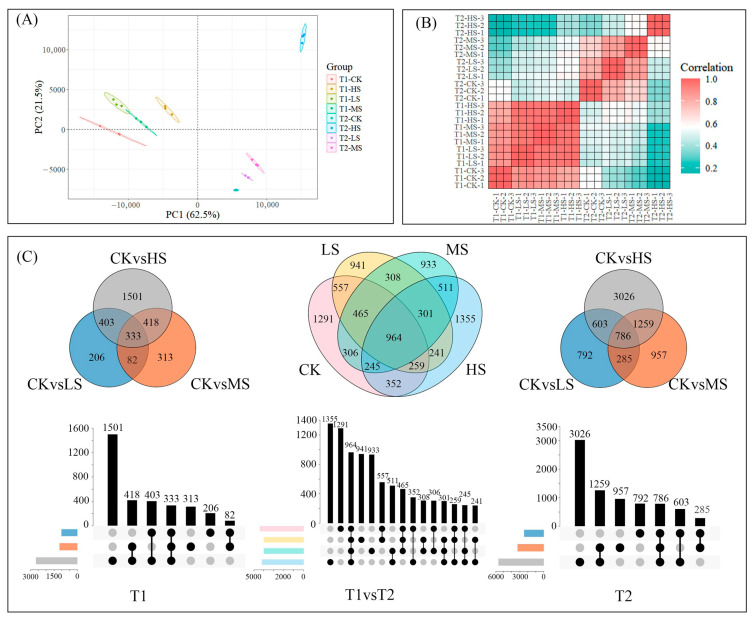
The PCA scatter plot, heatmap of correlation, and the number of DEGs in the transcriptomic profile of *C. paliurus* leaf samples. (**A**) The PCA scatter plot, where each point represents different salt stress biological replicates. (**B**) Heatmap of correlation coefficients DEGs. The red rectangles indicate strong correlations between samples, while the blue rectangles denote weak correlations. (**C**) Venn diagrams and column charts illustrating DEGs between samples from different salt stress levels and sampling times. The same color circles represent the same treatment. T1 and T2 denote the two sampling times, which correspond to 15 and 30 days after treatment, respectively. CK, LS, MS and HS indicate the non-, low-, mid-, and high-salinity treatments.

Approximately one billion high-quality clean reads were generated from 24 cDNA libraries derived from *C. paliurus* leaves after transcriptome data quality control analysis. The mapping rates for individual libraries ranged from 80.26% to 81.13%. Principal component analysis (PCA) revealed that PC1 and PC2 explained 62.5% and 21.5% of the variance in gene expression, respectively (Figure 2A), indicating distinct transcriptional profiles under different saline stress conditions. Hierarchical clustering further highlighted distinct correlations between samples collected at the two sampling times (Figure 2B). Notably, samples from T1 exhibited stronger correlations across different salt concentrations, while those from T2 showed weaker correlations, suggesting that gene expression patterns are influenced by the duration of salt treatment.

Transcriptome profiling enabled the identification of differentially expressed genes (DEGs) among samples subjected to varying salt concentrations at each sampling time (Figure 2C). Comparing with different salt treatments at the same sampling time, the greatest number of DEGs difference was found between CK and HS groups (ranging from 1501 to 3026 genes), while the smallest DEGs were observed between CK and LS treatments (ranging from 206 to 792 genes). Additionally, when comparing two sampling times, HS samples exhibited a higher number of DEGs (1355 specific genes) compared to CK samples (1291 specific genes). These results indicate that increasing salt concentrations could act as a stronger driver of transcriptomic changes, as evidenced by the greater number of DEGs associated with higher salt stress levels.

### 2.3. GO and KEGG Enrichment Analyses

**Figure 3:**
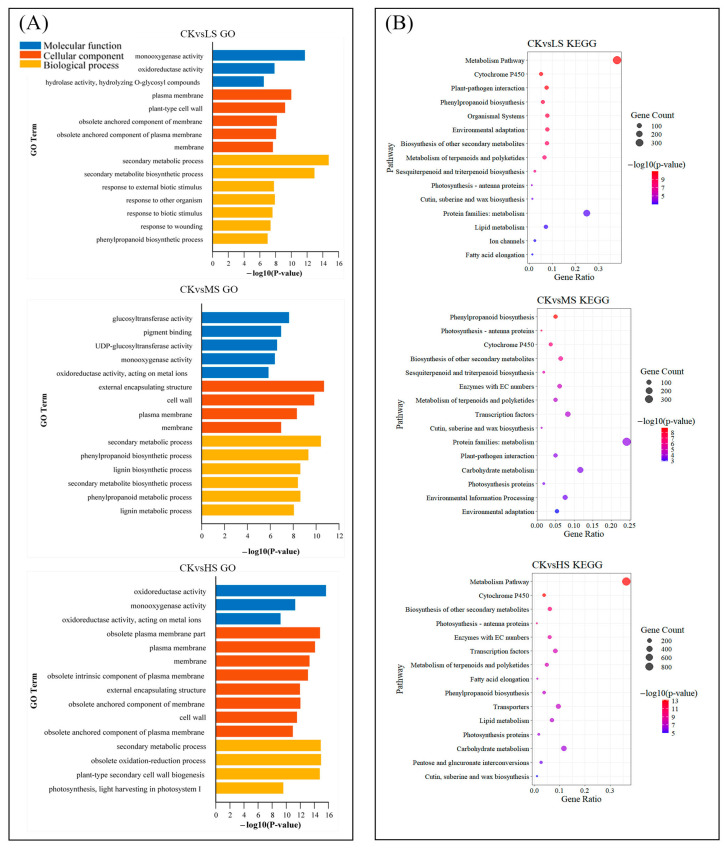
Gene Ontology (GO) enrichment bar plot and Kyoto Encyclopedia of Genes and Genomes (KEGG) bubble plot of DEGs in T1 (sampling 15 days after treatment). (**A**) The color of the bars represents different GO term categories: blue for molecular function, red for cellular component, and yellow for biological process. The length of the bars corresponds to −log10(*p*-value). (**B**) The size of the bubbles represents the gene count, from small to large, and the color gradient from blue to red reflects increasing −log10(*p*-value). CK, LS, MS and HS indicate the non-, low-, mid-, and high-salinity treatments.

The functions of DEGs were annotated using the Gene Ontology (GO) and categorized into three main classifications: cellular component, molecular function, and biological process. Across all comparison groups, 15 GO terms were significantly enriched (*p* < 0.05), encompassing all three categories (Figure 3A).

As anticipated, the DEGs were primarily enriched in the response to oxidoreductase activity, monooxygenase activity, plasma membrane, and secondary metabolic processes in *C. paliurus* (Figure 3A). The analysis of LS revealed that DEGs were predominantly enriched in monooxygenase activity, plasma membrane, and secondary metabolic processes. In the context of low-salt stress, *C. paliurus* primarily exhibits a response characterised by the activation of defence mechanisms and secondary metabolic pathways. Interestingly, the HS treatment exhibited a markedly greater number of membrane-associated GO terms compared to LS and MS, whereas LS exhibited a greater enrichment of GO terms related to stimulus response relative to the other two treatments.

The Kyoto Encyclopedia of Genes and Genomes (KEGG) enrichment analysis across different salt stress treatments further highlights key metabolic pathways that are differentially impacted by treatment time and increasing salt concentrations (Figure 3B). Notably, *C. paliurus* exhibited significant enrichment in cytochrome P450 pathways, as well as in cutin, suberin, and wax biosynthesis processes under LS treatment. It suggests that the activation of these metabolic pathways contributes to the reinforcement of multiple defense mechanisms against salt stress. In response to salinity stress, the DEGs involved in the phenylpropanoid biosynthesis and biosynthesis of other secondary metabolites pathways were found to be enriched in *C. paliurus*. Interestingly, pathways like lipid metabolism and fatty acid elongation were also enriched under high salinity, which may correlate with membrane stabilization during osmotic stress, indirectly supporting amino acid transport and metabolic adjustments.

### 2.4. Identification of WGCNA Modules Associated with Amino Acid Accumulation

**Figure 4:**
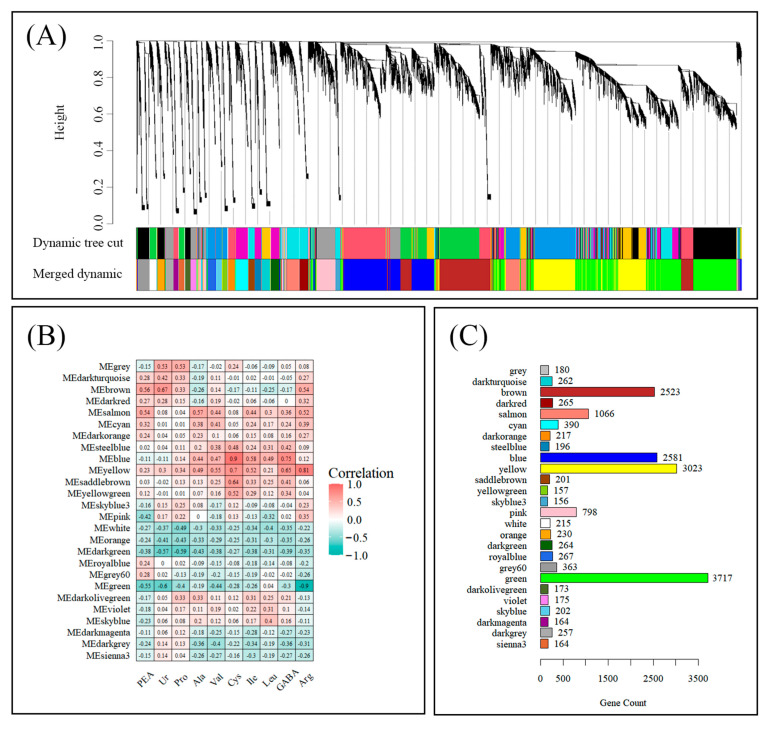
The WGCNA of transcriptomic and metabolome data. modules associated with amino acid accumulation. (**A**) Hierarchical clustering dendrogram of expressed genes, showing module classification generated by WGCNA. (**B**) Heatmap illustrating the correlation between module eigengenes and amino acid levels, with red indicating positive correlations and green indicating negative correlations. (**C**) Different colored rectangles represent gene numbers contained in each module.

Weighted gene co-expression network analysis (WGCNA) was conducted on 18,206 genes (coefficient of variation > 0.5), resulting in the identification of 26 distinct modules (Figure 4A). The relationship between each module’s gene expression profile and amino acid content was subsequently analyzed (Figure 4B). Among them, the yellow module exhibited a strong positive correlation with all amino acids, with Arg showing the highest Pearson correlation coefficient (r = 0.81), which also ranked third in abundance among total amino acids. Additionally, the green module displayed a significant negative correlation with most amino acids, particularly with Arg (r = −0.90). Gene numbers within each module ranged from 156 to 3717. Five modules (brown, salmon, blue, yellow, and green) contained more than 1000 genes, with 3023 and 3717 genes in the yellow and green modules, respectively (Figure 4C).

These two modules were further analyzed using KEGG and GO enrichment. Notably, genes within the yellow module were significantly enriched in glycine, serine and threonine metabolism. KEGG analysis further indicated enrichment in pathways related to plant hormone signal transduction, signal transduction and environmental information processing. GO analysis revealed a significant enrichment of genes involved in responses to abiotic stimulus and response to stress in yellow modules (Figure S1A). In contrast, a KEGG pathway analysis of the green module genes revealed an enrichment in pathways involved in carbohydrate metabolism, photosynthesis, antenna proteins, and protein families. Furthermore, the analysis of GO terms indicated that genes associated with photosynthetic processes were predominantly represented in the green module (Figure S1B).

Additionally, comparison of module eigengene expression profiles under saline conditions revealed contrasting trends between the yellow and green modules (Figure S1C). Specifically, the eigengenes in the yellow module exhibited an upward trend in expression with increasing salt concentrations, while those in the green module demonstrated a downward trend in expression in accordance with the salt concentration gradient.

### 2.5. Construction of Gene Co-Expression Networks

Based on the module connectivity, the top 15 genes with the highest weight values and the corresponding transcription factors (TFs) in the yellow and green modules were selected, respectively, for gene co-expression networks construction. Among the genes with the highest weight values in the yellow module, there were 8 TFs, and 16 were found in the green module.

**Figure 5:**
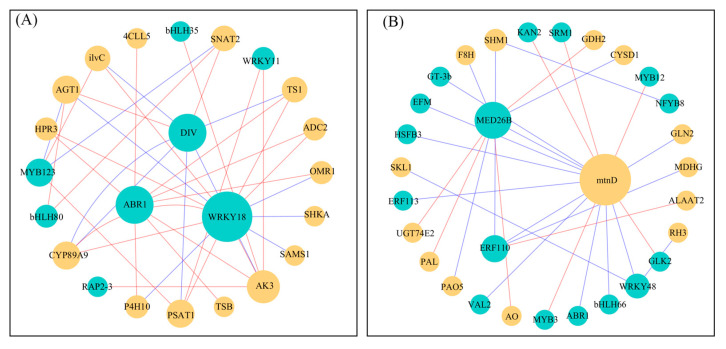
Gene co-expression networks constructed based on highly correlated TFs and structural genes involved in amino acid metabolism. Network visualization of yellow (**A**) and green (**B**) modules genes. In both networks, yellow circles denote core structural genes, while green circles represent TFs, with node size proportional to their connectivity. Edges between nodes are colored based on correlation type: red for positive correlations and green for negative correlations.

The 8 TFs identified in the yellow module (Figure 5A, Table S3) include DIV (*CpaF1st26414*), ABR1 (*CpaF1st22778*), WRKY18 (*CpaF1st30702*), RAP2-3 (*CpaF1st32079*), bHLH80 (*CpaF1st42893*), MYB12 (*CpaF1st36132*), bHLH35 (*CpaF1st31088*), and WRKY11 (*CpaF1st43219*). It revealed that the potential regulatory roles of these TFs modulated multiple structural genes involved in amino acid metabolic pathways by means of co-expression analysis, including genes encoding threonine synthase (TS1), arginine decarboxylase (ADC2), and tryptophan synthase (TSB). Of particular interest is the observation that the majority of these genes exhibited elevated levels of expression in MS and HS in T1 and CK in T2, as exemplified by *CpaF1st14666*, *CpaF1st33930*, and *CpaF1st31323*. Interestingly, certain genes demonstrated high levels of expression in the CK group across both T1 and T2, such as *CpaF1st02514*, *CpaF1st26414* and *CpaF1st42893*, although there are also some TFs that are highly expressed in the salt stress group, such as *CpaF1st30702* and *CpaF1st36132*. In T1, the expression levels of numerous genes, including *CpaF1st40193*, *CpaF1st46762*, and *CpaF1st22778*, exhibited an upward trend in response to increasing salt concentrations (Figure S2A).

As demonstrated in Figure 5B, the 16 TFs identified in the green module are DIVARICATA (*CpaF1st17187*), MED26B (*CpaF1st07247*), ERF110 (*CpaF1st24534*), WRKY48 (*CpaF1st41211*), ABR1 (*CpaF1st35052*), EFM (*CpaF1st24406*), KAN2 (*CpaF1st19634*), MYB12 (*CpaF1st32033*), NFYB8 (*CpaF1st46088*), GLK2 (*CpaF1st22007*), bHLH66 (*CpaF1st27322*), MYB114 (*CpaF1st04714*), VAL2 (*CpaF1st37128*), ERF113 (*CpaF1st02745*), HSFB3 (*CpaF1st07160*), and GT-3B (*CpaF1st09175*). The co-expression network has been demonstrated to indicate the presence of a number of structural genes associated with amino acid metabolism, including cysteine synthase (CYSD1), glutamine synthetase (GLN2) and glutamate dehydrogenase (GDH2). Notably, there are a number of genes that are highly expressed in both T1 and T2 CK groups, such as *CpaF1st05405*, *CpaF1st21395*, and *CpaF1st17187.* HS treatment resulted in a decline in the expression levels of certain genes as the sampling time progressed such as *CpaF1st06750*, *CpaF1st24406*, and *CpaF1st37128*. (Figure S2B).

### 2.6. Gene Expression and Metabolite Accumulation in Carbohydrate and Amino Acid Metabolism Process

**Figure 6:**
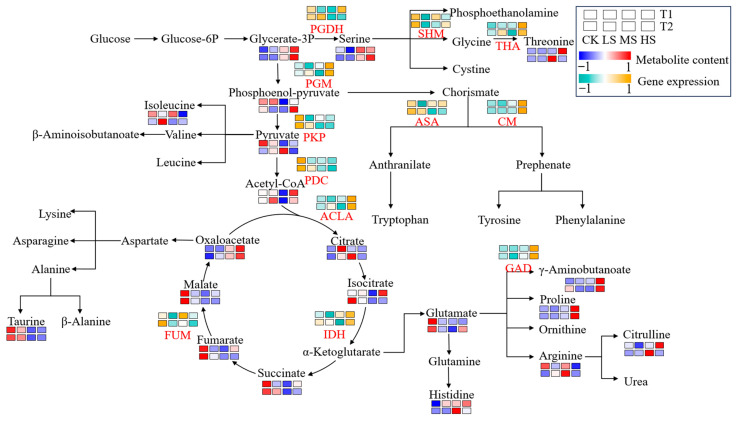
Amino acid biosynthesis pathway and corresponding heatmaps of gene expression and metabolite abundance. Rectangles colored from green to yellow indicate gene expression intensity from low to high, while rectangles shifting from blue to red depict increasing metabolite abundance. Refer to Figure 1 for abbreviations CK, LS, MS and HS. Enzyme abbreviations: PGDH, Phosphoglycerate dehydrogenase; SHM, serine hydroxymethyltransferase; THA, L-threonine aldolase; PGM, phosphoglycerate mutase; PKP, pyruvate kinase isozyme; ASA, anthranilate synthase alpha; PDC, pyruvate decarboxylase; CM, chorismate mutase; ACLA, ATP citrate-lyase; GAD, glutamate decarboxylase; IDH, isocitrate dehydrogenase; FUM, fumarate hydratase. T1 and T2 denote the two sampling times, which correspond to 15 and 30 days after treatment, respectively. CK, LS, MS and HS indicate the non-, low-, mid-, and high-salinity treatments.

In accordance with the results of WGCNA, several structural genes associated with the metabolism pathways of amino acids and carbohydrate were identified within the yellow and green modules. Concurrently, we identified the relationships between gene expression and a variety of metabolites and then constructed a regulatory map (Figure 6).

The expression levels of genes in control encoding pyruvate kinase isozyme (PKP, *CpaF1st47087*) and pyruvate decarboxylase (PDC, *CpaF1st37253*) were up-regulated in comparison to the salt stress treatment. From the metabolite profiles, the control treatment exhibited increased accumulation of certain metabolites, primarily from the TCA cycle, including malate, fumarate and succinate, as well as some amino acids, such as taurine and glutamate, in contrast to the salt stress treatment. Conversely, the expression levels of certain key amino acid synthase genes, such as L-threonine aldolase (THA, *CpaF1st32727*), chorismate mutase (CM, *CpaF1st24104*) and glutamate decarboxylase (GAD, *CpaF1st01888*), were found to be elevated in the HS treatment, which may have promoted the accumulation of the corresponding amino acids in *C. paliurus*. Remarkably, the analysis revealed that the salt-stressed treatment exhibited higher levels of most amino acids and some carbohydrates, including serine, threonine, GABA, proline, citrulline, glycerate-3P, citrate, and oxaloacetate, in comparison to the CK. Furthermore, the expression levels of certain enzymes associated with carbohydrate metabolism, including phosphoglycerate mutase (PGM, *CpaF1st00428*), ATP citrate-lyase (ACLA, *CpaF1st01376*) and isocitrate dehydrogenase (IDH, *CpaF1st02899*), were also found to be elevated in the salt-stressed treatment. These observations indicate that salt stress may potentially influence the accumulation of metabolites and the activities of related enzymes, which in turn may affect amino acid and carbohydrate metabolism.

## 3. Discussion

Amino acids, found widely distributed in *C. paliurus* leaves, are crucial to growth and environmental adaptation. Previous research has demonstrated that salt stress influences the accumulation of amino acids in plants [23]. However, there is a scarcity of studies conducted to explore the effects of different salt stresses on amino acid accumulation in *C. paliurus*. The present study utilized *C. paliurus* in a hydroponic experiment, comparing the amino acid contents of *C. paliurus* leaves at four saline concentrations (CK, LS, MS, and HS) at two sampling times (T1 and T2). By integrating transcriptomic data with amino acid content, gene co-expression networks were constructed. It facilitated the identification of key genes and TFs involved in amino acid accumulation. Furthermore, combined metabolomic analysis suggested that the dynamic adjustment of carbohydrate and amino acid partitioning in *C. paliurus* leaves constituted a pivotal mechanism for adapting to varying salinity concentrations.

### 3.1. Salinity Effects on Amino Acids Accumulation

In addition to their medicinal and nutritional value, amino acids have a significant impact on plant environmental adaptation by enhancing stress resistance mechanisms and facilitating defense responses to abiotic challenges. Plants frequently accumulate protective substances such as amino acids in order to alleviate damage when confronted with stress conditions. Previous studies have shown that proline is an essential osmoregulatory substance, and its content tends to increase in response to salt stress [24,25,26]. Similarly, in the present study, the content of proline in T1 *C. paliurus* leaves increased with increasing salt concentration. Notably, the proline content of HS was observed to be approximately 3 times that of LS (Table S1). Glutamate is involved in response to abiotic stresses and glutamate metabolism was activated under such conditions, thereby enhancing stress resistance through the production of proline and GABA [27]. Compared to CK, glutamate content was higher under salt stress (Table S1), which was different from the results of Zhang’s [28]. Interestingly, the branched-chain amino acids (BCAAs), including isoleucine, valine and leucine, undergo a decrease in accumulation under salt stress. In contrast, threonine, the precursor of 2-ketobutyrate synthesis, has been observed to increase (Table S1). This observation contradicts the findings reported by Sun [29]. This discrepancy may be explained by the observation that the isoleucine precursor, 2-ketobutyrate, is synthesized by the key enzyme threonine deaminase in plants, and that the synthesis of this enzyme is feedback-inhibited by all three BCAAs [30].

### 3.2. Salinity Effects on Gene Expression

The PCA conducted in this study demonstrated that both salt stress concentration and sampling time had a pronounced effect on gene expression in *C. paliurus* leaves (Figure 2A). This finding corroborates the previous perspective that plants employ distinct molecular responses under salt stress to regulate transcriptional processes [31,32]. For instance, samples collected at T1 showed a high degree of similarity in gene expression between CK and HS treatments (correlation coefficient > 0.80), whereas this relationship was notably weaker at T2 (correlation coefficient < 0.50). It indicates that by the time of T2, *C. paliurus* had adapted to salt stress compared to the results of T1. This may be related to a decrease in lysine concentration which is one of the defense mechanisms against abiotic stress (Figure 2B). Furthermore, an increase in salinity was found to result in an enhancement of the number of DEGs (Figure 2C).

Phenylpropanoids are a class of organic compounds synthesized by plants through the process of phenylpropanoid biosynthesis using aromatic amino acids as precursors [33] and have been shown to make a difference in the scavenging of ROS and improving the salt resistance of the plant [34]. KEGG results demonstrated that phenylpropanoid biosynthesis pathway was enriched in all salt treatments (Figure 3). Correspondingly, the accumulation of phenylalanine (one of aromatic amino acids) decreased under salt stress (Table S1), which is in agreement with the results of *Solanum lycopersicum* [35]. This indicates that the genes related to the phenylpropanoid biosynthesis pathway in *C. paliurus* seedlings are highly responsive to salt stress, potentially through the activation of phenylpropanoid biosynthesis. It involves the utilization of phenylalanine to generate phenylpropanoids, which function as antioxidants by scavenging excess ROS produced by individual plants under salt stress.

### 3.3. Regulated Gene Network of Amino Acids Biosynthesis

Specialized compound biosynthesis is subject to the influence of a variety of environmental factors, which act in a manner that is dependent on the specific set of TFs involved in the regulation of the relevant gene expression [36]. WGCNA is an algorithm used to extract module information from gene expression data by clustering similar expression patterns genes into modules and analyzing the specific characteristics of each module [37]. Today, WGCNA is the most widely adopted method for identifying patterns of gene correlation. It is increasingly applied in plant research to uncover the distinct co-expression modules in plants under salt stress [38]. In this study, we constructed two gene co-expression networks in order to investigate the potential biosynthesis mechanisms of amino acid in *C. paliurus* leaves under salt stress (Figure 4).

Within the yellow module co-expression network, which demonstrated a positive correlation with amino acid biosynthesis and accumulation, several structural genes and TFs involved in the amino acid metabolic pathway were identified (Figure 5A). For example, we found the TS1 gene in the co-expression network. This gene produces threonine synthase, which is an important enzyme in the production of threonine [39]. This network suggests that TS1 expression is regulated by complex TFs such as SRM1, WRKY18 and ABR1. Of particular interest is the observation that two TS genes (*CpaF1st04693* and *CpaF1st46762*) within the co-expression network demonstrated significant up-regulation in a short time in HS samples (Figure S2A), which is the same as the results observed in grape [40]. However, the highest threonine accumulation was found in MS, whic may be attributed to the up-regulation of the expression of OMR1 (Figure S2A), which encodes threonine dehydratase, a key enzyme that catalyses the conversion of threonine to 2-ketobutyrate [41].

A phenylalanine ammonia lyase (PAL) gene was identified in the green module co-expression network (Figure 5B) that exhibited a negative correlation with biosynthesis and accumulation of amino acids. PAL is the first rate-limiting enzyme of the phenylpropane metabolic pathway and makes a great difference in the synthesis of secondary metabolites such as flavonoids and phenolic acids [42,43,44]. As previously mentioned, phenylpropane metabolism uses aromatic amino acids as substrates. PAL expression was up-regulated in HS (Figure S2B), and a large number of aromatic amino acids were synthesised as secondary metabolites to enhance salinity resistance in *C. paliurus* seedlings, which concurrently resulted in the accumulation of phenylalanine in HS being reduced (Table S1), which is in agreement with the results of *Ginkgo biloba* [33].

As demonstrated in the regulatory map, GABA and proline exhibited the highest concentrations in *C. paliurus* leaves under HS (Figure 6). It has been demonstrated that GABA has the capacity to augment tolerance to abiotic stress by means of regulating ion transport, intracellular pH, facilitating ROS and scavenging activating antioxidant systems [16,45,46,47]. It is also notable that proline, one of the pivotal osmoregulatory compounds within plant cells, plays an important role in maintaining osmotic equilibrium under salt stress [48,49,50,51]. Intriguingly, the content of Glutamate exhibited a contrasting pattern to that of GABA and proline (high in CK and low in HS), while the expression of GAD exhibited a synchronous pattern with that of GABA. This may be related to the fact that both GABA and proline are converted from Glutamate, and in fact, GABA can be synthesised from glutamate by glutamic acid decarboxylase (GAD) catalysed glutamate decarboxylation, which has been termed the GABA shunt [52,53].

## 4. Materials and Methods

### 4.1. Plant Materials and Treatments

Seeds of *C. paliurus* (Jinzhongshan No. 11 family) were originally collected from Jinzhongshan County (24°58′ N, 110°09′ E), Guangxi Autonomous Region, China. To break dormancy and enhance germination efficiency, the seeds underwent stratification treatment and were pretreated with exogenous gibberellin A3 (GA3), as described by Fang [54]. The germinated seeds were planted in nonwoven containers filled with a mixed growth medium composed of perlite, topsoil, decomposed poultry manure, and peat in a 2:2:2:4 volume ratio. The substrate had the following composition: 74.3 g/kg organic matter, 72.4 g/kg total N, 9.57 g/kg total K, 2.21 g/kg total P, and a pH of 6.45. Seedlings were irrigated every two days. After three months of growth, seedlings of uniform size were transplanted into 50 L polypropylene containers filled with half-strength Hoagland’s nutrient solution (pH 6.0 ± 0.1) and cultivated in a greenhouse of Baima Experimental Base of Nanjing Forestry University (31°35′ N, 119°09′ E). After a two-week acclimation period, the seedlings were maintained in the same nutrient solution for subsequent experimental treatments.

Referring to the previous study of YAO [55], four levels of NaCl concentration were set up: control (CK, 0 mM), low-salinity (LS, 0.15%), mid-salinity (MS, 0.30%), and high-salinity (HS, 0.45%). For each treatment, 24 seedlings with a height of 50–55 cm and ground diameter of 3.7–4.2 mm were used. A completely randomized block design was employed to arrange the seedlings. Each treatment contained 3 biological replicates which were composed of 8 seedlings each. During the experimental period, the daytime air temperature ranged from 25–32 °C, and the nighttime temperature ranged from 17–22 °C. The relative air humidity varied between 65% and 80%.

### 4.2. Sample Collection, Amino Acids Extraction and Determination

Leaf samples of *C. paliurus* were collected at two time points, 15 days (T1) and 30 days (T2), following salt stress treatments. Three seedlings were randomly selected for each treatment, and six mature leaves from different positions on each tree were sampled. Subsequently, the leaves were subjected to immediate freezing in liquid nitrogen, followed by storage at −80 °C for further analysis.

The extraction of amino acids was performed by hydrolyzing 50 mg of leaf tissue in 5 mL of 6 M HCl for 24 h at 110 °C, following the method of Zhou [56]. Amino acid concentrations were determined using a Sykam S433D amino acid analyzer (Sykam, Munich, Germany) by ion exchange chromatography, coupled with post-column derivatization with ninhydrin. The elution gradient consists of eluent A (0.12 M sodium citrate buffer at pH 3.45), elution buffer B (0.20 M citric acid-sodium citrate buffer at pH 10.85), and buffer D (0.50 M sodium hydroxide), with a flow rate of 0.45 mL/min, as follows: 0–10 min, 0% B; 10–16 min, 15% B; 16–22 min, 20% B; 22–26 min, 33% B; 26–30 min, 80% B; 30–41 min, 100% B; 41–45 min, 100% D. The separated amino acids were detected using spectrophotometry at wavelengths of 440 nm (proline) and 570 nm (other amino acids). They were calibrated using a standard amino acid mixture from Sykam GmbH Company (Munich, Germany), containing taurine (Tau), tyrosine (Tyr), aspartic acid (Asp), O-phosphorylethanolamine (PEA), threonine (Thr), phenylalanine (Phe), urea (Ur), serine (Ser), glutamic acid (Glu), glycine (Gly), valine (Val), proline (Pro), histidine (His), alanine (Ala), asparagine (Asn), tryptophan (Trp), citrulline (Cit), cystine (Cys), isoleucine (Ile), leucine (Leu), β-alanine (β-Ala), γ-aminobutyric acid (GABA), 3-aminoisobutyric acid (BABA), lysine (Lys), ornithine (Orn), and arginine (Arg). Amino acid score (AAS) and essential amino acid index (EAAI) were calculated using the following equations, respectively:

$$AAS=\frac{a_{p}}{a_{s}}$$

$$EAAI=\sqrt[{n}]{{Lys}_{1p}/{Lys}_{1s}\times {Tyr}_{2p}/{Tyr}_{2s}\times \cdot \cdot \cdot \times {His}_{np}/{His}_{ns}}\times 100\%$$

where *a* is an essential amino acid (EAA), *p* is the test protein, *s* is the reference protein and *n* is the number of amino acids entering into the calculation [56].

PCA was conducted on the identified amino acids components by using the R package [57].

### 4.3. Metabolomics Analysis

Metabolites for each sample were extracted according to the method outlined by Guo [58]. Briefly, 50 mg of freeze-dried leaf powder was extracted with 1 mL of extraction solvent (acetonitrile: methanol: water in a 2:2:1 ratio), containing 0.1 mg/L lidocaine as an internal standard. The homogenization and ultrasonic extraction procedures were repeated in triplicate, followed by a 60-min incubation period at 20 °C. Subsequently, the samples were centrifuged at 10,000× *g* for 10 min at 4 °C, and the supernatants were filtered. Metabolite analysis was carried out using an ultra-high-performance liquid chromatography (UHPLC) system (Santa Clara, CA, USA) equipped with a UPLC HSS T3 column and interfaced with a Q Exactive Orbitrap mass spectrometer (Waltham, MA, USA). Instrumental parameters were consistent with those reported by Guo [58]. For metabolomic profiling, peaks were annotated using OSI-SMMS software (Version 1.0) based on an internal MS/MS spectral library. The annotated metabolites were then mapped to relevant metabolic pathways using the KEGG database.

### 4.4. Transcriptomics Analysis

Total RNA from each sample was extracted according to the protocol outlined by Guo [58], using the Trizol reagent kit (Carlsbad, CA, USA). After verifying RNA quality, mRNA was isolated and fragmented to prepare for cDNA library construction. A total of 24 cDNA libraries were generated, representing two sampling time points under four salt stress conditions, each with three biological replicates. Sequencing was performed using the Illumina HiSeq2500 platform to produce paired-end reads. Clean reads were aligned to the *C. paliurus* reference genome (https://ngdc.cncb.ac.cn/gwh/Assembly/26380/show (accessed on 30 October 2024)) using the Hisat2 alignment tool. Differentially expressed genes (DEGs) were identified using the DESeq2 package (version 1.44.0) in R based on thresholds of false discovery rate (FDR) < 0.05 and fold change (|FC|) > 2. Subsequent functional enrichment of DEGs was conducted using Gene Ontology (GO) and Kyoto Encyclopedia of Genes and Genomes (KEGG) pathway analyses, with significance set at *p* < 0.05.

### 4.5. Weighted Gene Co-Expressed Network Analysis (WGCNA)

Genes were identified across all 24 samples and filtered based on gene expression, with a coefficient of variation greater than 0.5. Transcript abundance was calculated in fragments per kilobase of transcript per million mapped reads (FPKM). Using the WGCNA (Version 1.73) R package, an adjacency matrix was constructed to assess the relationships between genes, with a threshold power of 10. Modules of co-expressed genes were identified using the dynamic tree cut algorithm, with parameters set to a merge cut height of 0.4 and a minimum module size of 150. For each module, the eigengene (the first principal component) was computed and used to calculate Pearson correlation coefficients with the concentrations of the ten most abundant amino acids in *C. paliurus* leaves. Modules showing statistically significant correlations (*p* < 0.05) were selected for downstream analysis. KEGG enrichment analysis was then performed on genes from the selected modules. From each key module, the 15 genes which own the highest weight values, along with their corresponding transcription factors (TFs), were identified. The resulting gene regulatory networks were constructed using Cytoscape (Version 3.10.0) [59].

### 4.6. Statistics Analysis and Visualisations

All statistical analyses and visualisations were performed using different R packages for R (Version 4.4.1), factoextra (Version 1.0.7) for PCA, ggplot2 (Version 3.5.1) for bubble plots and pheatmap (Version 1.0.12) for heatmaps. TBtools software (Version 2.142) was used to visualize venn diagrams and GO enrichment analysis [60].

## 5. Conclusions

This study demonstrates that the amino acid composition in *C. paliurus* leaves varies significantly under different sampling times and salt concentrations. Some amino acids, such as proline, act as osmotic regulators and accumulate to higher levels under salt stress. In contrast, other amino acids, such as phenylalanine and glutamate, are converted into other protective compounds under salt stress, resulting in lower accumulation. Consequently, the amino acids present in the leaves of *C. paliurus* under salt stress have the potential to enhance its salt resistance through both direct and indirect mechanisms. Our findings elucidate the amino acid response mechanisms in *C. paliurus* under salt stress, thereby providing a foundation for its genetic enhancement and cultivation in affected regions. Future investigations would benefit from expanding both the sample size and the range of metabolites analyzed.

## Data Availability

The Illumina raw sequencing profiles were submitted to the NCBI BioProject database under number PRJNA700136.

## Supplementary Materials

The following supporting information can be downloaded at https://www.mdpi.com/article/10.3390/ijms262110444/s1.
