# Supplementary material for: Multi-Omics Analysis Reveals the Regulation of Amino Acid Biosynthesis in Cyclocarya paliurus Leaves Under Salt Stress
Source: Int J Mol Sci. 2025 Oct 27;26(21):10444. doi: 10.3390/ijms262110444 (PMC12608903; doi:10.3390/ijms262110444)
Supplement: Supplementary file 1 [file ijms-26-10444-s001.zip › ijms-3932629-supplementary.pdf]

Supplementary Materials:

Table S1. Amino acid content in different samples from different treatments.

Times	Compounds	Retention time (min)	Amino acid content							
			CK		LS		MS		HS	
			mg/g	%	mg/g	%	mg/g	%	mg/g	%
T1	1.Tua	4.85	0.013±7.48E-04	0.6	0.008±9.73E-04	1.1	0.012±7.40E-04	0.7	0.018±2.31E-03	1.1
	2.PEA	5.96	0.047±2.63E-03	2.1	0.04±2.54E-03	5.4	0.077±4.31E-03	4.3	0.049±3.92E-03	3
	3.Ur	6.88	0.797±8.31E-03	36.4	0.237±3.54E-02	31.9	0.738±3.37E-02	41.4	0.86±4.68E-02	52.3
	4.Asp	16.98	0.014±1.58E-03	0.6	0.008±6.60E-04	1.1	0.015±6.50E-04	0.8	0.011±1.15E-03	0.7
	5.Thr	23.11	0.028±1.84E-03	1.3	0.016±1.13E-03	2.2	0.043±2.00E-03	2.4	0.026±4.32E-03	1.6
	6.Ser	25.04	0.012±2.01E-03	0.5	0.003±6.60E-04	0.4	0.008±7.40E-04	0.4	0.009±1.11E-03	0.5
	7.Asn	27.93	0.033±2.07E-03	1.5	0.023±1.50E-03	3.1	0.066±4.05E-03	3.7	0.044±2.69E-03	2.7
	8.Glu	29.93	0.003±1.38E-03	0.1	0.002±3.40E-04	0.3	0.004±8.50E-04	0.2	0.003±3.20E-04	0.2
	9.Pro	39.45	0.085±1.21E-02	3.9	0.026±4.00E-03	3.5	0.064±7.21E-03	3.6	0.076±4.04E-03	4.6
	10.Gly	42.03	0.011±1.42E-03	0.5	0.007±4.50E-04	0.9	0.009±5.00E-04	0.5	0.01±8.10E-04	0.6
	11.Ala	43.93	0.14±1.65E-02	6.4	0.06±5.60E-03	8.1	0.091±8.40E-03	5.1	0.072±6.80E-03	4.4
	12.Cit	45.56	-	-	-	-	0.006±1.28E-03	0.3	0.009±1.08E-03	0.5
	13.Val	51.43	0.049±3.88E-03	2.2	0.016±2.00E-03	2.2	0.028±3.98E-03	1.6	0.018±2.50E-03	1.1
	14.Cys	56.47	0.437±3.90E-02	19.9	0.134±2.00E-02	18.1	0.285±3.28E-02	16.1	0.232±6.75E-03	14.1
	15.Ile	62.94	0.074±4.68E-03	3.4	0.027±2.04E-03	3.6	0.041±2.68E-03	2.3	0.03±2.83E-03	1.8
	16.Leu	64.52	0.098±1.09E-02	4.5	0.031±8.07E-03	4.2	0.057±6.89E-03	3.2	0.048±3.53E-03	2.9
	17.Tyr	67.59	0.008±3.10E-04	0.4	0.009±2.50E-04	1.2	0.012±1.22E-03	0.7	0.007±4.50E-04	0.4
	18.Phe	71.17	0.023±2.37E-03	1	0.01±1.25E-03	1.3	0.016±3.08E-03	0.9	0.009±1.63E-03	0.5
	19.β-Ala	74.09	0.005±5.05E-04	0.2	0.002±8.15E-04	0.3	0.004±6.11E-04	0.2	0.002±4.77E-04	0.1
	20.BABA	74.94	0.003±7.30E-04	0.1	-	-	0.002±3.00E-04	0.1	0±1.90E-04	0
	21.GABA	77.01	0.175±1.07E-02	8	0.046±4.57E-03	6.2	0.122±1.89E-02	6.9	0.066±6.03E-03	4
	22.His	81.51	0.005±5.40E-04	0.2	0.003±3.80E-04	0.4	0.005±8.30E-04	0.3	0.005±1.80E-04	0.3
	23.Trp	88.89	0.015±1.01E-03	0.7	0.007±5.80E-04	0.9	0.013±2.50E-03	0.7	0.008±9.00E-04	0.5
	24.Orn	92.19	0.002±9.60E-04	0.1	0.001±2.30E-04	0.1	0.002±4.10E-04	0.1	0.002±4.10E-04	0.1
	25.Lys	93.42	0.015±2.60E-03	0.7	0.005±5.10E-04	0.7	0.01±1.55E-03	0.6	0.006±1.95E-03	0.4
	26.Arg	100.91	0.104±1.28E-02	4.7	0.021±7.12E-03	2.8	0.052±1.26E-02	2.9	0.026±3.57E-03	1.6
	Total		2.195±8.07E-02	100	0.742±9.33E-03	100	1.781±1.72E-02	100	1.645±4.14E-02	100
T2	1.Tua	4.85	0.005±8.96E-04	0.2	0.009±6.06E-04	0.4	0.013±1.05E-03	0.5	0.014±1.51E-03	0.5
	2.PEA	5.96	0.065±5.04E-03	2.4	0.044±3.65E-03	1.7	0.081±3.43E-03	3.3	0.085±5.54E-03	3.1

3.Ur	6.88	0.733±2.80E-02	27.1	0.931±2.49E-02	36.3	0.753±3.64E-02	30.8	1.203±1.66E-01	42.9
4.Asp	16.98	0.006±6.26E-04	0.2	0.014±3.78E-03	0.5	0.008±5.59E-04	0.3	0.015±1.52E-03	0.5
5.Thr	23.11	0.026±1.77E-03	1	0.024±1.79E-03	0.9	0.036±2.84E-03	1.5	0.029±9.45E-04	1
6.Ser	25.04	0.005±1.08E-03	0.2	0.014±2.52E-03	0.5	0.011±9.10E-04	0.5	0.018±2.10E-03	0.6
7.Asn	27.93	0.026±5.61E-03	1	0.117±1.09E-02	4.6	0.08±8.00E-03	3.3	0.17±1.80E-02	6.1
8.Glu	29.93	0.008±1.79E-04	0.3	0.038±5.01E-03	1.5	0.008±1.01E-03	0.3	0.049±1.08E-02	1.7
9.Pro	39.45	0.074±6.03E-03	2.7	0.086±2.89E-03	3.4	0.069±3.06E-03	2.8	0.087±9.85E-03	3.1
10.Gly	42.03	0.015±6.92E-04	0.6	0.011±9.35E-04	0.4	0.008±9.44E-04	0.3	0.008±8.32E-04	0.3
11.Ala	43.93	0.124±9.84E-03	4.6	0.109±9.93E-03	4.3	0.155±1.16E-02	6.4	0.082±5.86E-03	2.9
12.Cit	45.56	-	-	-	-	-	-	0.008±1.93E-03	0.3
13.Val	51.43	0.056±7.96E-03	2.1	0.031±5.99E-03	1.2	0.056±1.25E-02	2.3	0.047±3.47E-03	1.7
14.Cys	56.47	0.904±1.10E-01	33.3	0.49±5.26E-02	19.2	0.405±1.03E-02	16.6	0.347±1.89E-02	12.5
15.Ile	62.94	0.076±5.68E-03	2.8	0.048±2.89E-03	1.9	0.073±7.44E-03	3	0.052±7.99E-03	1.9
16.Leu	64.52	0.086±9.77E-03	3.2	0.046±1.86E-02	1.8	0.081±9.17E-03	3.3	0.053±1.34E-02	1.9
17.Tyr	67.59	0.005±7.12E-04	0.2	0.013±1.34E-03	0.5	0.011±2.76E-03	0.5	0.006±1.23E-03	0.2
18.Phe	71.17	0.015±2.01E-03	0.6	0.009±7.50E-04	0.4	0.008±1.62E-03	0.3	0.018±2.63E-03	0.6
19.β-Ala	74.09	0.006±5.05E-04	0.2	0.005±7.90E-04	0.2	0.005±9.20E-04	0.2	0.004±6.91E-04	0.1
20.BABA	74.94	0.004±9.57E-04	0.1	0.003±9.60E-04	0	-	-	-	-
21.GABA	77.01	0.233±9.75E-03	8.6	0.144±2.03E-02	5.6	0.185±1.04E-02	7.6	0.123±6.09E-03	4.5
22.His	81.51	0.005±7.44E-04	0.2	0.007±7.02E-04	0.3	0.006±1.06E-03	0.2	0.008±9.28E-04	0.3
23.Trp	88.89	0.012±1.50E-03	0.4	0.026±4.15E-03	1	0.016±2.18E-03	0.7	0.009±1.10E-03	0.3
24.Orn	92.19	0.003±8.35E-04	0.1	0.005±9.80E-04	0.2	0.002±6.34E-04	0.1	0.004±3.60E-04	0.1
25.Lys	93.42	0.017±1.46E-03	0.6	0.019±1.37E-03	0.7	0.021±2.31E-03	0.9	0.015±9.70E-04	0.5
26.Arg	100.91	0.2±1.52E-02	7.3	0.32±2.55E-02	12.5	0.349±4.92E-02	14.3	0.349±7.04E-03	12.4
Total		2.709±1.17E-01	100	2.564±4.97E-02	100	2.44±6.40E-02	100	2.804±1.94E-01	100

Amino acid abbreviations: taurine (Tau), O-phosphorylethanolamine(PEA), urea (Ur), aspartic acid (Asp), threonine (Thr), serine (Ser), asparagine (Asn), glutamic acid (Glu), proline (Pro), glycine (Gly), alanine (Ala), citrulline (Cit), valine (Val), cystine (Cys), isoleucine (Ile), leucine (Leu), tyrosine (Tyr), phenylalanine(Phe), β-alanine (β-Ala), 3-aminoisobutyric acid (BABA), γ-aminobutyric acid (GABA), histidine (His), tryptophan(Trp) ornithine (Orn), lysine (Lys), and arginine (Arg). T1, T2 represented two sampling times, e.g. 15 and 30 days after treatments.CK, LS, MS and HS represented four levels of NaCl treatment (control, 0.15 %, 0.30 % and 0.45 %, m/v) in this study, respectively.

Table S2. Rotated component matrix.

	PC1	PC2	PC3	PC4	PC5
1.Tua	-0.071	-0.247	0.309	0.113	0.155
2.PEA	0.140	-0.131	0.255	0.284	-0.282
3.Ur	0.202	-0.270	0.124	-0.160	0.011
4.Asp	0.029	-0.277	0.039	-0.065	0.469
5.Thr	0.108	-0.085	0.270	0.368	0.159
6.Ser	0.179	-0.304	0.028	-0.025	0.106
7.Asn	0.137	-0.344	-0.102	0.033	-0.147
8.Glu	0.150	-0.288	-0.200	-0.163	-0.148
9.Pro	0.237	-0.143	0.128	-0.186	0.194
10.Gly	0.159	0.186	0.060	-0.372	0.050
11.Ala	0.240	0.151	0.058	0.256	0.116
12.Cit	-0.087	-0.281	0.299	-0.141	-0.033
13.Val	0.263	0.096	0.160	0.118	-0.172
14.Cys	0.237	0.200	-0.016	-0.225	-0.109
15.Ile	0.265	0.170	0.135	0.096	-0.041
16.Leu	0.204	0.186	0.273	0.094	0.144
17.Tyr	0.010	-0.055	-0.332	0.405	0.355
18.Phe	0.102	0.013	0.313	-0.194	0.230
19.β-Ala	0.280	0.109	-0.048	0.037	-0.002
20.BABA	0.177	0.211	-0.043	-0.267	0.274
21.GABA	0.280	0.178	0.067	0.034	-0.047
22.His	0.192	-0.279	-0.034	-0.075	-0.110
23.Trp	0.181	-0.017	-0.359	0.082	0.365
24.Orn	0.222	-0.121	-0.272	-0.200	-0.052
25.Lys	0.293	0.012	-0.131	0.164	-0.071
26.Arg	0.248	-0.133	-0.174	0.133	-0.263

Refer to Table S1 for amino acid abbreviations.

Table S3. Reference table of genes and symbols.

Yellow module		Green module	
Gene ID	Symbols	Gene ID	Symbols
CpaF1st14666	AGT1	CpaF1st04884	mtnD
CpaF1st02514	SNAT2	CpaF1st33875	CYSD1
CpaF1st04693	TS1	CpaF1st05405	GLN2
CpaF1st40193	OMR1	CpaF1st06750	RH3
CpaF1st24298	AK3	CpaF1st34299	AO
CpaF1st33930	PSAT1	CpaF1st21395	PAO5
CpaF1st46762	TS1	CpaF1st11202	UGT74E2
CpaF1st32983	P4H10	CpaF1st24280	SKL1
CpaF1st00869	CYP89A9	CpaF1st07133	F8H
CpaF1st48360	CYP89A9	CpaF1st33398	SHM1
CpaF1st46300	HPR3	CpaF1st31265	GDH2
CpaF1st39472	ilvC	CpaF1st45123	MDHG
CpaF1st43704	4CLL5	CpaF1st04774	ALAAT2
CpaF1st03076	ADC2	CpaF1st36150	PAL
CpaF1st31323	SHKA	CpaF1st17187	DIV
CpaF1st43875	SAMS1	CpaF1st07247	MED26B
CpaF1st39150	TSB	CpaF1st24534	ABR1
CpaF1st26414	DIV	CpaF1st41211	WRKY48
CpaF1st22778	ABR1	CpaF1st35052	ABR1
CpaF1st30702	WRKY18	CpaF1st24406	EFM
CpaF1st32079	RAP2-3	CpaF1st19634	KAN2
CpaF1st42893	bHLH80	CpaF1st32033	MYB12
CpaF1st36132	MYB123	CpaF1st46088	NFYB8
CpaF1st31088	bHLH35	CpaF1st22007	GLK2
CpaF1st43219	WRKY11	CpaF1st27322	bHLH66
		CpaF1st04714	MYB3
		CpaF1st37128	VAL2
		CpaF1st02745	ERF113
		CpaF1st07160	HSFB3
		CpaF1st09175	GT-3B

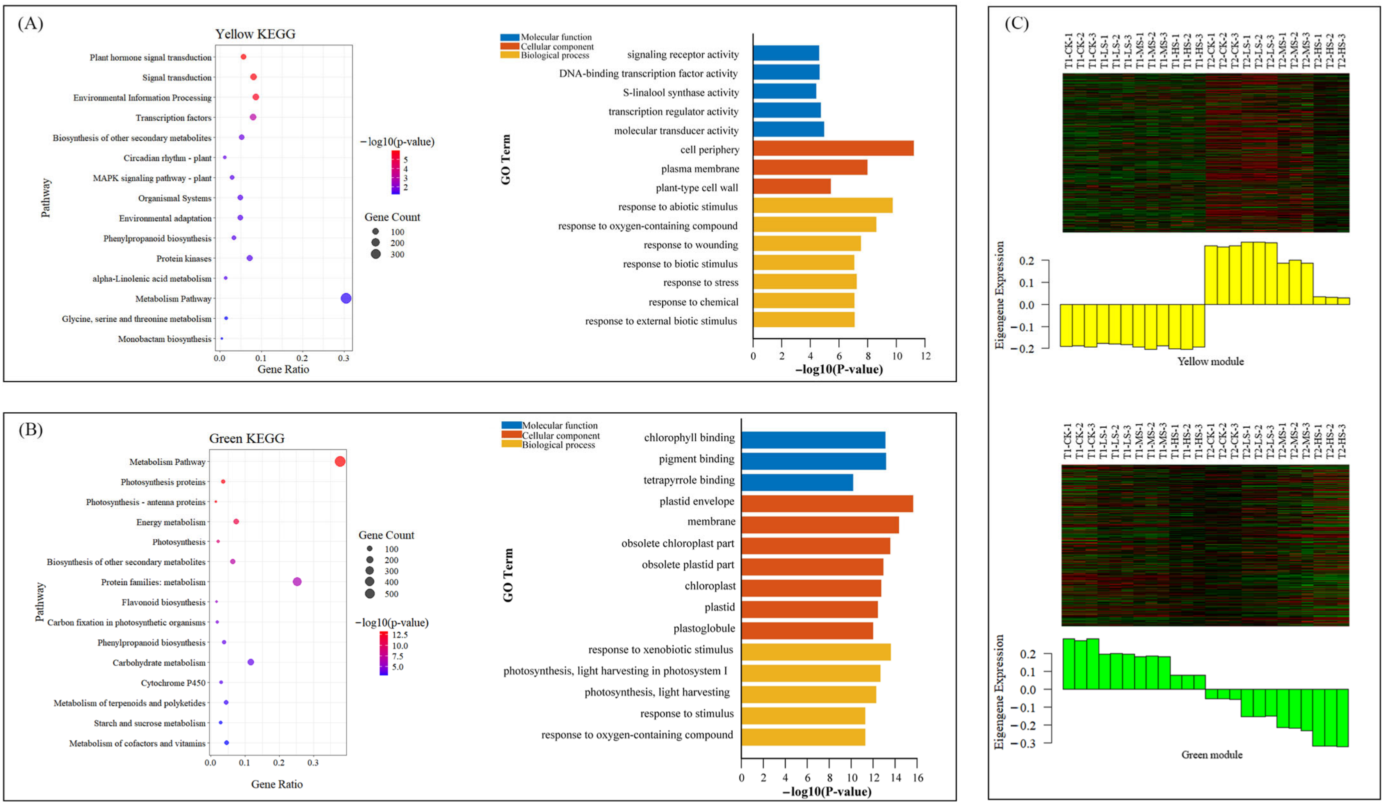

Figure S1. KEGG and GO enrichment analysis of genes in yellow (A) and green (B) modules. (C) Eigengenes expression profiles of yellow module and green module. CK, LS, MS and HS, indicate the non-, low-, mid-, and high- salinity treatments.

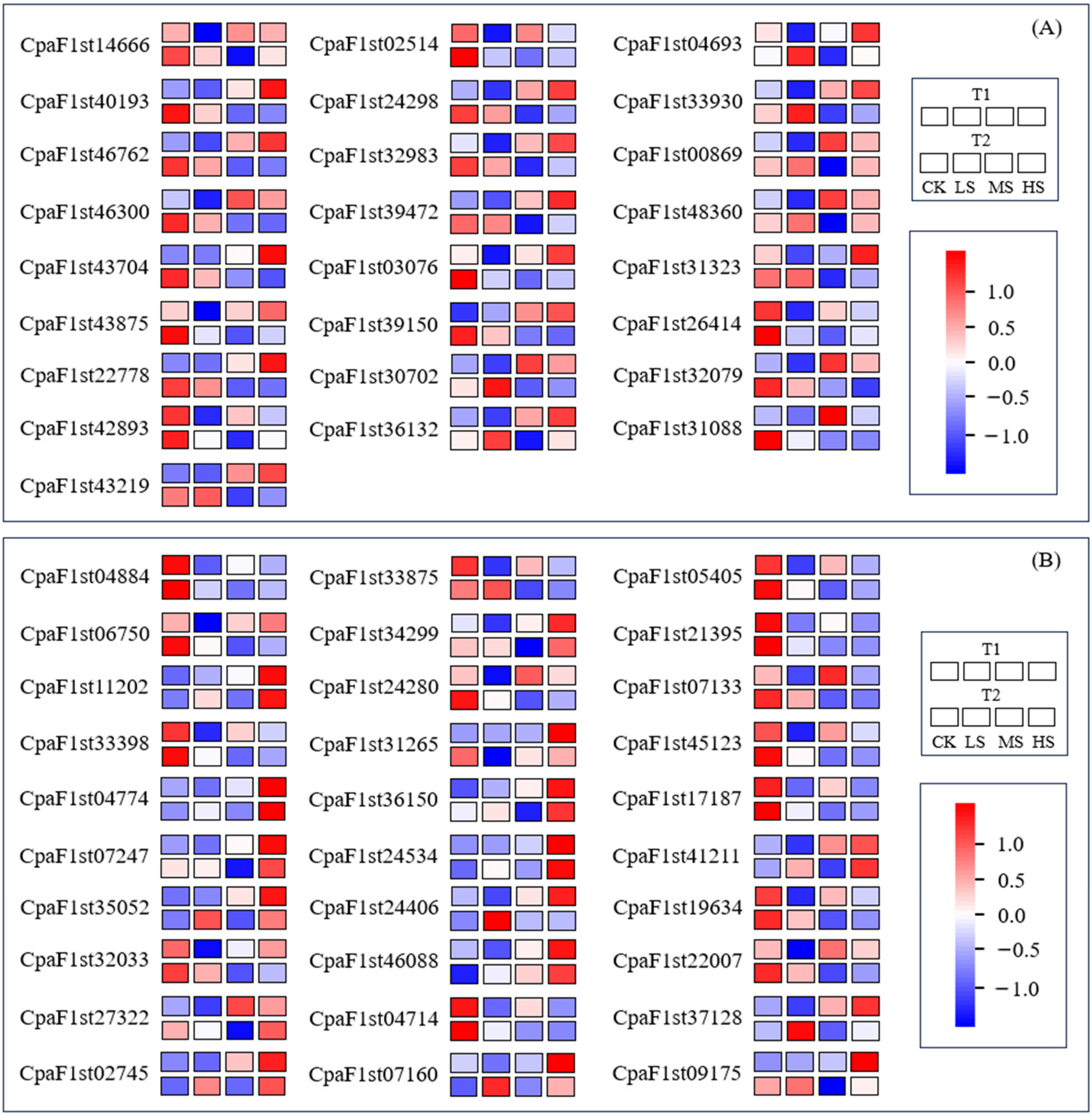

Figure S2. Heat maps of the expression of genes encoding transcription factors and key enzymes in the yellow (A) and green (B) modules. A change from blue to red indicates gene expression from low to high. CK, LS, MS and HS, indicate the non-, low-, mid-, and high- salinity treatments.
